# TRAM-01: A phase 2 study of trametinib for pediatric patients with neurofibromatosis type 1 and plexiform neurofibromas

**DOI:** 10.1093/neuonc/noag112

**Published:** 2026-05-19

**Authors:** Dorsa Sadat Kiaei, Maxime Caru, Valérie Larouche, Emma Clini, Deepika Pugalenthi Saravanan, Sarah Lippé, Yves Théoret, Jean-Claude Décarie, Uri Tabori, Cynthia Hawkins, Benjamin Ellezam, Luis H Ospina, Léandra Desjardins, Marie-Élaine Métras, Serge Sultan, Édith Cantin, Marie-Ève Routhier, Chantal Mailloux, Marie-Claude Bertrand, Karim Estephan, Tara McKeown, Stéphanie Vairy, Geneviève Legault, Samuele Renzi, Éric Bouffet, Vijay Ramaswamy, Hallie Coltin, Lucie Lafay-Cousin, Juliette Hukin, Mélanie Alves, Tiffany Rent, Craig Erker, Nada Jabado, Mathieu Dehaes, Sébastien Perreault

**Affiliations:** Department of Neurosciences, University of Montreal, CHU Sainte-Justine, Montreal, Canada; Department of Pediatrics, Division of Hematology and Oncology, Pennsylvania State Health Children’s Hospital, Hershey, Pennsylvania, USA; Department of Pediatrics, Division of Hematology-Oncology, CHU de Québec—Université Laval, Québec, Canada; Department of Neurosciences, University of Montreal, CHU Sainte-Justine, Montreal, Canada; Department of Pediatrics, Division of Hematology and Oncology, Pennsylvania State Health Children’s Hospital, Hershey, Pennsylvania, USA; Department of Psychology, Université de Montréal, Montréal, Canada; Centre de recherche Azrieli, CHU Sainte-Justine, Montreal, Canada; Department of Pharmacology, CHU Sainte Justine, Montreal, Canada; Medical Imaging Department, CHU Sainte-Justine, Montreal, Canada; Department of Pediatrics, University of Toronto, Toronto, Ontario, Canada; Division of Hematology Oncology, The Hospital for Sick Children, Toronto, Ontario, Canada; Department of Pathology, Hospital for Sick Children, Toronto, Canada; Department of Pathology, CHU Sainte Justine, Montreal, Canada; Department of Ophthalmology, CHU Sainte Justine, Montreal, Canada; Department of Psychology, Université de Montréal, Montréal, Canada; Centre de recherche Azrieli, CHU Sainte-Justine, Montreal, Canada; Department of Psychology, Université de Montréal, Montréal, Canada; Department of Psychology, CHU de Québec—Université Laval, Québec, Canada; Department of Psychology, CHU de Québec—Université Laval, Québec, Canada; Department of Psychology, Université de Montréal, Montréal, Canada; Department of Psychology, Université de Montréal, Montréal, Canada; Department of Neurosciences, University of Montreal, CHU Sainte-Justine, Montreal, Canada; Division of Hematology Oncology, The Hospital for Sick Children, Toronto, Ontario, Canada; Department of Pediatrics, University of Toronto, Toronto, Ontario, Canada; Department of Pediatrics, Division of Pediatric Hematology-Oncology, CHU Sherbrooke, Québec, Canada; Department of Pediatrics, Division of Neurology, Montreal Children’s Hospital, Québec, Canada; Department of Pediatrics, Division of Hematology-Oncology, CHU de Québec—Université Laval, Québec, Canada; Department of Pediatrics, University of Toronto, Toronto, Ontario, Canada; Division of Hematology Oncology, The Hospital for Sick Children, Toronto, Ontario, Canada; Department of Pediatrics, University of Toronto, Toronto, Ontario, Canada; Division of Hematology Oncology, The Hospital for Sick Children, Toronto, Ontario, Canada; Department of Hematology-Oncology, CHU Sainte-Justine, Montreal, Canada; Department of Hematology-Oncology, Alberta Children’s Hospital, Alberta, Canada; Department of pediatrics, Division of Hematology-Oncology, B.C. Children’s Hospital, University of British Columbia, Canada; Department of Neurosciences, University of Montreal, CHU Sainte-Justine, Montreal, Canada; Division of Pediatric Hematology-Oncology, IWK Health Centre, Halifax, Nova Scotia, Canada; Division of Pediatric Hematology-Oncology, IWK Health Centre, Halifax, Nova Scotia, Canada; Department of Pediatrics, Dalhousie University, Halifax, Nova Scotia, Canada; Departments of Pediatrics and Human Genetics, Division of Pediatric Hematology-Oncology, McGill University Health Center, Montreal, Canada; Centre de recherche Azrieli, CHU Sainte-Justine, Montreal, Canada; Department of Radiology, Radio-oncology and Nuclear Medicine, University of Montreal, Montreal, Canada; Department of Neurosciences, University of Montreal, CHU Sainte-Justine, Montreal, Canada

**Keywords:** clinical trial, MEK inhibitor, neurofibromatosis type 1, plexiform neurofibroma, trametinib

## Abstract

**Background:**

Plexiform neurofibromas (PNs) are observed in up to 50% of patients with neurofibromatosis type 1 (NF1). Trametinib has been used widely to treat PNs but limited data has been reported on its efficacy within a clinical trial. We conducted a phase 2 trial with trametinib for patients with NF1 and inoperable PNs.

**Methods:**

Patients with NF1 and inoperable PNs aged ≥1 month to ≤25 years when starting trametinib were eligible. Patients received trametinib once daily orally continuously for a maximum of 18 cycles of 28 days. Patients completed patient-reported outcome measures. Response evaluations were performed after cycle 3, 6, 12 and at the end of treatment and then every 6 months. The PN volumes were centrally quantified using a semi-automatic 3D segmentation method.

**Results:**

Forty-six patients were recruited. The median age was 11.1 years (range 0.7-19.8). The median baseline tumor volume was 104.6 ml (range 16.3 to 650.1 ml). Volumetric assessment demonstrated an overall response rate (volume decrease of ≥20%) in 47.8% (22/46) of patients. Median volume change was −19.3% (range −80.5 to 3.5). Forty-one patients (89.1%) completed the planned treatment. The 3 years progression free survival and the event free survival were respectively 73.1% (95% CI 53.3%-85.5%) and 47.1% (95% CI 28.8-63.5).

**Conclusions:**

We report outcomes and volumetric response of PNs treated with trametinib within a phase 2 trial. Based on these results, trametinib is effective and overall well tolerated.

Key Points

- Trametinib demonstrated an overall response rate of 47.8% in patients with plexiform neurofibroma.
- Treatment was generally well tolerated, with most adverse events being grade 1 or 2.
- Patient-reported outcome assessments showed improvement during treatment.

Importance of the Study

We report the results of the first large cohort of patients with NF1 and PN treated prospectively on study with trametinib. Therapy was discontinued after 18 months for all patients. This provides a unique opportunity to understand long-term outcomes once treatment is stopped, a critical aspect not addressed in previous clinical trials.

We demonstrate the efficacy with an ORR of 47.8% with a median volume change of −19.3%. Overall, treatment was well tolerated with mainly grade 1 and 2 AEs. Less than a third of patients required a dose reduction and only three patients discontinued treatment before the planned duration of the trial. Significant improvements in patient-reported outcomes were observed during treatment. Inspite of most patients having shrinkage during treatment almost a third of patients (32.6%) required a new intervention following discontinuation. The median EFS was 35 months and the median time to restart treatment after discontinuation was 10 months.

Plexiform neurofibromas (PNs) are tumors associated with neurofibromatosis type 1 and observed in up to 50% of patients.^1^ Surgical resection of these slow growing lesions is often limited due to location and morbidity associated with surgery. The management of PNs has changed over the last decade with the use of MEK inhibitors (MEKi). Selumetinib was approved in several countries for treatment of unresectable and symptomatic PNs given its benefits on tumor volume reduction and improvement of functional outcome.^2^ This MEKi is giving twice daily and currently available in form of tablets and sprinkle formulation. Recently, mirdametinib was approved by the FDA for the treatment of children and adults with plexiform neurofibromas. For this indication, mirdametinib is administered twice daily on a schedule of three weeks on, followed by one week off.^3^ Mirdametinib is available in form of capsules and dispersible tablets. Another study also reported clinical responses with binimetinib, which is administered twice daily using a tablet formulation or liquid formulation.^4–5^ Currently, there are no data supporting the use of a MEKi over another based on schedule or administration. However, while many patients with PNs worldwide have been treated with trametinib due to ease of access and liquid formulation for young children, there is limited data on its efficacy within a clinical trial.^6^ We conducted a phase 2 trial with trametinib which included one arm for patients with NF1 and inoperable PNs. We report the final results of the treatment phase and preliminary results of the follow-up phase for the PNs cohort.

## Methods

### Study Design and Population

The TRAM-01 trial is an open label phase 2 clinical trial using trametinib for patients with NF1 and inoperable PN or refractory pediatric low-grade gliomas with MAPK/ERK pathway activation (NCT03363217).^6^ The study was conducted in accordance with the Declaration of Helsinki, and the study was approved by the institutional review board on May 14, 2018 (IRB-CHU Sainte-Justine MP-21-2018-1742). Written informed consent and assent were obtained for all patients.

Patients were enrolled in seven Canadian pediatric centers. For the PN cohort, inclusion criteria included: age ≥1 month to ≤25 years when starting trametinib and unresectable PN, either causing morbidity and/or growing with the potential to cause morbidity. Patients who received a MEK inhibitor (MEKi) prior to trial were eligible. Other eligibility and exclusion criteria are detailed in Table SA1. The primary objective for the PN cohort was to evaluate the response rate to trametinib.

PN were categorized based on location and aspect. Distinct nodular lesions were defined and categorized centrally as well-demarcated, encapsulated-appearing, ≥3 cm lesions.^7^

### Therapy

Patients received trametinib as a single daily dose of 0.025 mg/kg (patients ≥6 years) or 0.032 mg/kg (patients <6 years) to a maximum of 2 mg per day. This dosing schedule is based on a previous phase 1 trial.^8^ Trametinib was available as liquid formulation or as oral tablets depending on patient weight or needs. Patients could receive a maximum of 18 continuous cycles of 28 days. Patients were discontinued from therapy for trametinib-related dose limiting toxicity (DLT) or progressive disease (PD) based on RECIST 1·1 evaluation by the local investigator(s).^9^ Dose modification and toxicity management are detailed in Data S1.

### Study Evaluation

Patients underwent MRI at designated time points: baseline (prior to treatment), after cycle 3, cycle 6, cycle 12, end of treatment and every 6 months for 36 months. Response evaluation was done by the treating team using RECIST 1.1 criteria.^9^ In the objective of comparing one-dimension responses with volumetric assessments, reduction of ≥10% and <30% (taking as reference the baseline or nadir sum of diameters) was considered as minor response. Assessment of PN volume was done centrally and independently using a semi-automatic algorithm.^10^ Partial response (PR) was defined as a ≥ 20% reduction in volume and progressive disease (PD) as a ≥ 20% increase in volume of the target PN compared to baseline or nadir. These response definitions are based on previous work from Dr. Widemann and collaborators.^11–13^ The best overall response rate (ORR) was defined as the best response during treatment.

### Safety Monitoring

Safety monitoring included physical examination and laboratory studies (including blood counts, liver function tests, lipase and creatine phosphokinase). Patients had an ophthalmology, electrocardiogram and echocardiogram evaluations after cycle 3, 6, 12 and end of treatment. Adverse events (AEs) were graded according to National Cancer Institute Common Terminology Criteria for Adverse Events v5.0.

### Patient-Reported Outcome (PROs) Measures

PROs were measured using the Pediatric Quality of Life Inventory (PedsQL) brain tumor module and generic core scales^14^^,^^15^ at designated time points: baseline, cycle 7, cycle 13, and end of treatment.

Age-specific versions of the questionnaires were used. Patients older than 5 years old completed the child self-report version, while one caregiver of children older than 2 years old completed the parent proxy-report version. French and English version of the validated questionnaires were used.^16^

The PedsQL questionnaires consist of 5-point Likert-scale questions (0 = never; 1 = almost; 2 = sometimes; 3 = often; 4 = almost always) where a high score indicates a good health-related quality of life.^14^ The scaling and scoring was conducted following the PedsQL recommendations (Data S2 for detailed method).

### Pharmacokinetic Analysis

Whole blood samples were collected at day 22, and before starting cycle 16 or at discontinuation of treatment whichever came first. Samples were assayed for trametinib serum level.

Trametinib was measured in plasma using a high-performance liquid chromatography (HPLC) system (Series 1200 Infinity) coupled to a tandem mass spectrometer (MS/MS 6460), both from Agilent Technologies Inc, Canada. Plasma calibration samples and three plasma quality control samples were prepared by adding the appropriate amount of trametinib (Toronto Research Chemicals, Canada) to blank plasma (Data S2 for detailed method).

### Statistical Analysis

Descriptive statistics are presented as median (minimum, maximum) for continuous variables and as count and proportion for categorical variables.

Waterfall and swimmer plots were produced to illustrate participant responses and treatment use over time.

Progression free survival and event free survival up to the last post-treatment follow-up were analyzed using Kaplan–Meier approach and displayed with Kaplan–Meier curves. Point estimates are presented along with the 95% confidence interval. An event was defined as either a progression of the lesion based on volumetric assessment compared to baseline or any intervention with the intent to treat the PN following discontinuation of trametinib such as surgery, re-introduction of a MEKi, or any other PN-directed systemic therapy.

Duration of response was defined as the delay between first occurrence of response during treatment and progressive disease (or last follow-up, if no progression occurred), censoring at the time anti-tumor therapies were resumed if applicable.

Association between response during treatment and baseline characteristics and serum level of trametinib were tested using Mann-Whitney test for continuous variables and chi-square test or Fisher’s exact test for categorical variables.

Treatment effects on PROs across the designated time points were examined using a RM-ANOVA. When significant effects were found, Bonferroni-adjusted post hoc tests were performed to identify differences between time points.

Statistical tests were conducted at the 0.05 significance level with SAS version 9.4 and IBM SPSS statistics, version 29.0.2.0 (IBM Corp). Data cut-off for evaluation was March 13, 2024.

## Results

### Patient Characteristics

Forty-seven patients were screened and 46 enrolled between August 16, 2018 and March 6 2022. The median duration of follow-up was 45.7 months (range 17.7-54.2 months).

Median age was 11.1 years (range 0.7-19.8 years) (Table 1). Thirty-four patients (73.9%) had never received treatment for their plexiform, whereas twelve (26.1%) received between 1 and 7 lines of treatment before enrolment. Seven patients (58.3%) were treated with imatinib, 4 (33.3%) received interferon, 4 (33.3%) underwent surgery and 2 (16.7%) were treated with trametinib (Data S3). The median PN volume at baseline was 104.6 ml (range 16.3-650.1 ml). Four patients out of 37 evaluable patients (10.8%) were considered to have progressive plexiform prior to enrolment. A total of 22 (47.8%) patients were symptomatic at baseline.

**Table 1.** Patient characteristics

Variable	Statistic
**Age at enrollment, years**	
N	46
Median	11.1
Age range	0.7-19.8
Age group (0 to <3 years old)	6 (13.0%)
Age group (3 to <12 years old)	21 (45.7%)
Age group (≥12 years old)	19 (41.3%)
**Sex**	
Male	22 (47.8%)
Female	24 (52.2%)
**Plexiform location**	
Head	17 (37.0%)
Neck	10 (21.7%)
Trunk	19 (41.3%)
Limb	9 (19.6%)
**With symptoms at presentation**	26 (56.5%)
Pain	11 (42.3%)
Ocular	9 (34.6%)
Weakness	7 (26.9%)
Urogenital	5 (19.2%)
Dysphagia	3 (11.5%)
Respiratory	3 (11.5%)
Other	2 (7.7%)
**Distinct nodular lesion plexiform**	10 (23.8%)
**Prior PN targeting medical therapies**	
None	34 (73.9%)
At least one	12 (26.1%)
**Tumor volume at baseline (ml)**	
*N*	46
Median	104.5
Range	16.3-650.1

### Plexiform Response

Forty-one (89.1%) completed the planned number of cycles (Median number of cycles 18 cycles, range 5-18 cycles).

**Figure 1.**
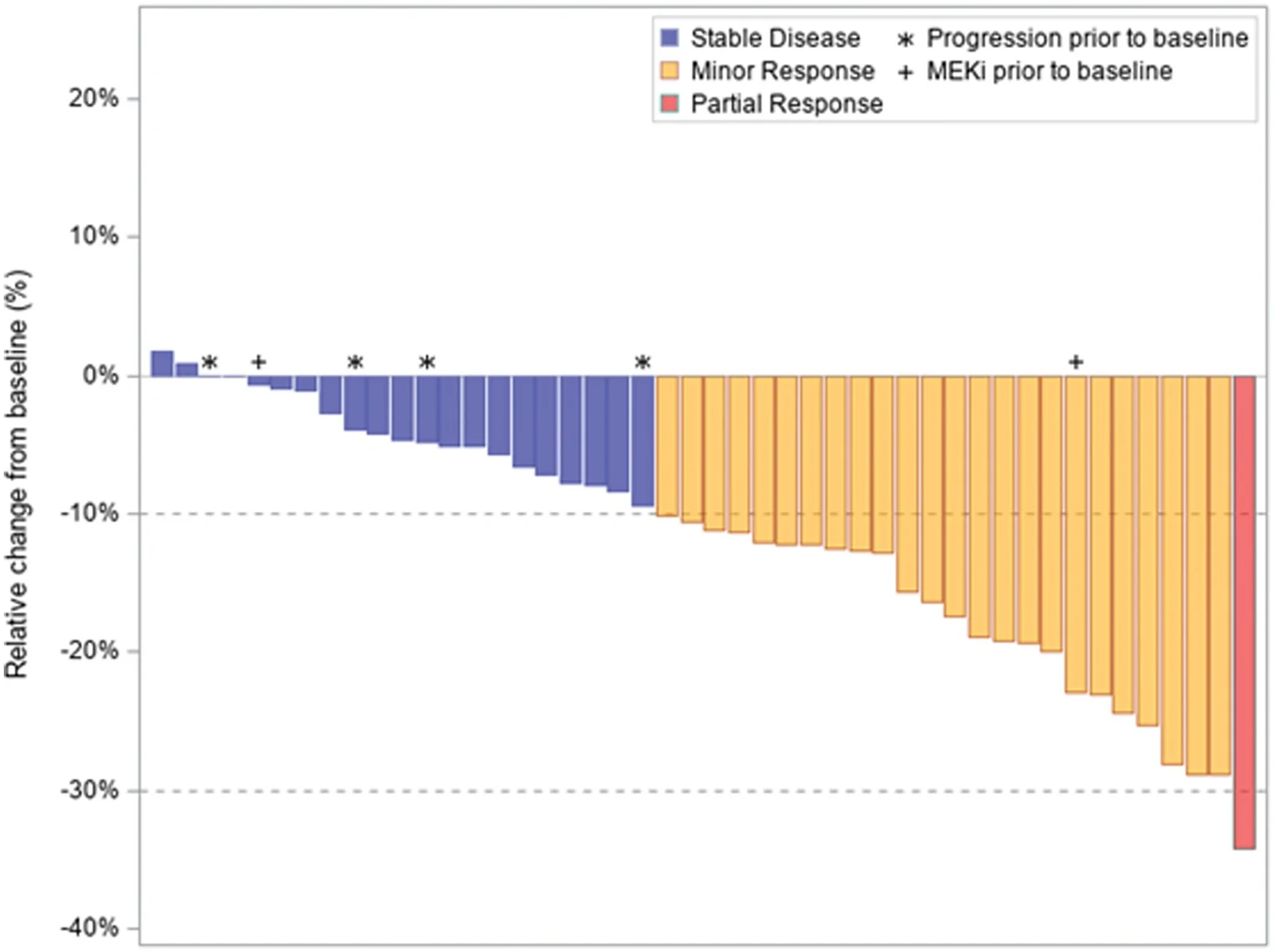
Waterfall plot: relative change from baseline to smallest observation during treatment (diameter) using the modified RECIST 1.1 criteria.

**Figure 2.**
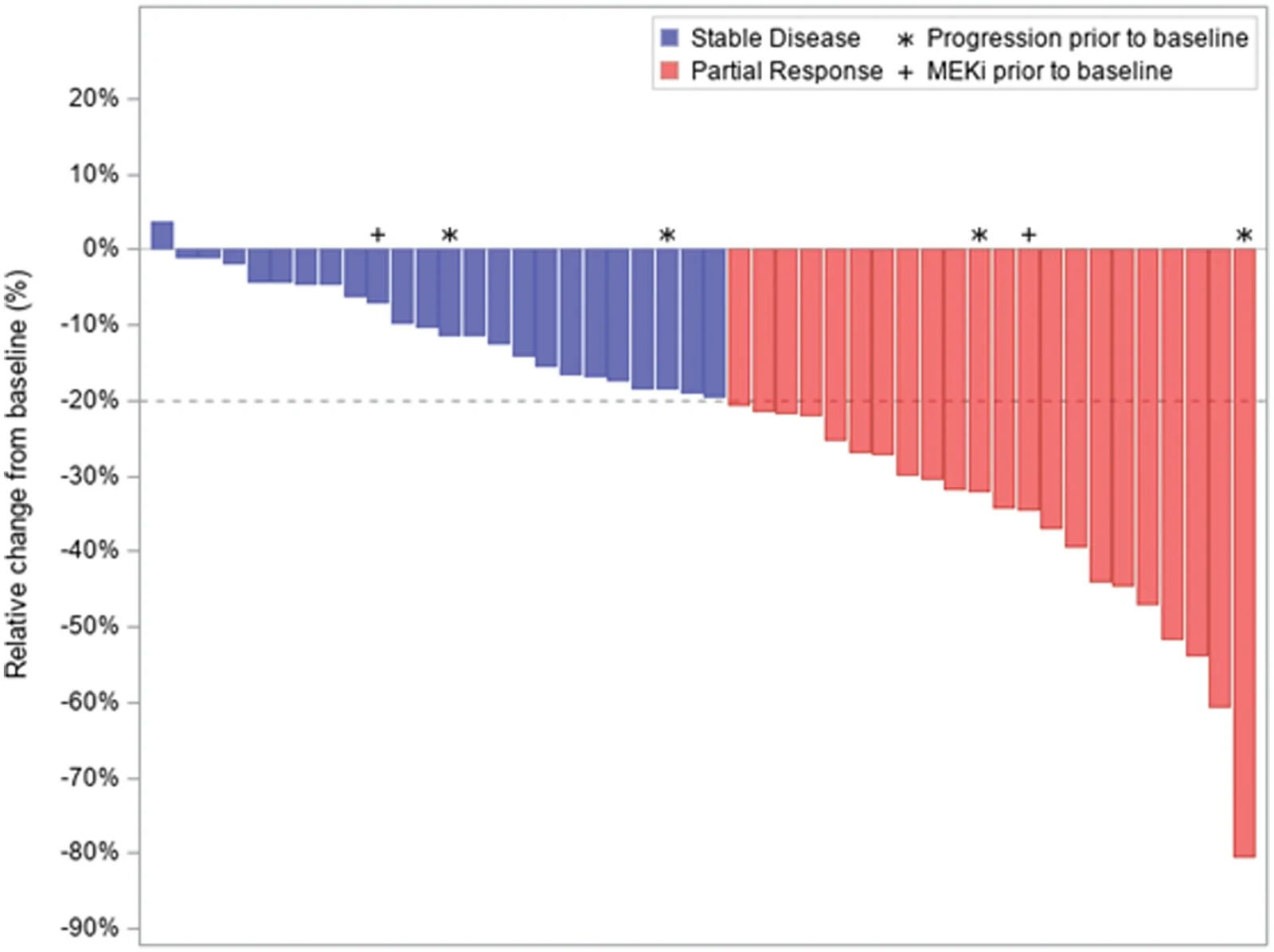
Waterfall plot: relative change from baseline to smallest observation during treatment using the volumetric analysis.

Based on RECIST 1.1 evaluation, the ORR was 2.2% with one patient achieving a partial response as best response during treatment and 45 patients (97.8%) had stable disease (Figure 1). When using PN volumetric evaluation compared to baseline the ORR was 47.8% with 22 patients achieving a partial response as best response during treatment, whereas 24 patients (52.2%) had stable disease (Figure 2). A total of 52 PN were considered target lesions. Of these, 26 (50%) achieved a volumetric partial response, while the remaining 26 (50%) were stable.

Median relative change from baseline to smallest volume during treatment was −19.3% (range −80.5 to 3.5). Median time to partial volumetric response when compared to baseline was 5.3 months (range 2.5-16.8). Median duration of response for volumetric evaluation was 14.2 months (range 3.0-44.8 months).

No association was found between volumetric response compared to baseline and age at baseline, sex, progression prior to enrolment or location. Median serum level of trametinib at cycle 1 was 11.6 ng/ml (range 2-29.2). No correlation was found between serum level and response.

Patients with distinct nodular lesions appeared to have a lower response rate compared to other patients (volumetric PR 20% vs 56.2% Fisher’s Exact Test *P* = .07) but without reaching a significant difference. Median age for DNL was 15.9 years (range 3.3-19.8) compared to 9.9 years for non-DNL (range 0.7-19.8) (*t* test *P* = .05).

When including patients with minor response [24 patients (52.2%)], the concordance response between one dimension measurement and volumetric assessment was 58.7%. There was discordance for 19 patients, eight patients (17.5%) with partial volumetric response were considered stable on modified RECIST evaluation and 11 (23.9%) patients were considered to show minor response but showed stable disease on volumetric measurements (Figure SA1 and Table SA2).

### Plexiform Outcome

**Figure 3.**
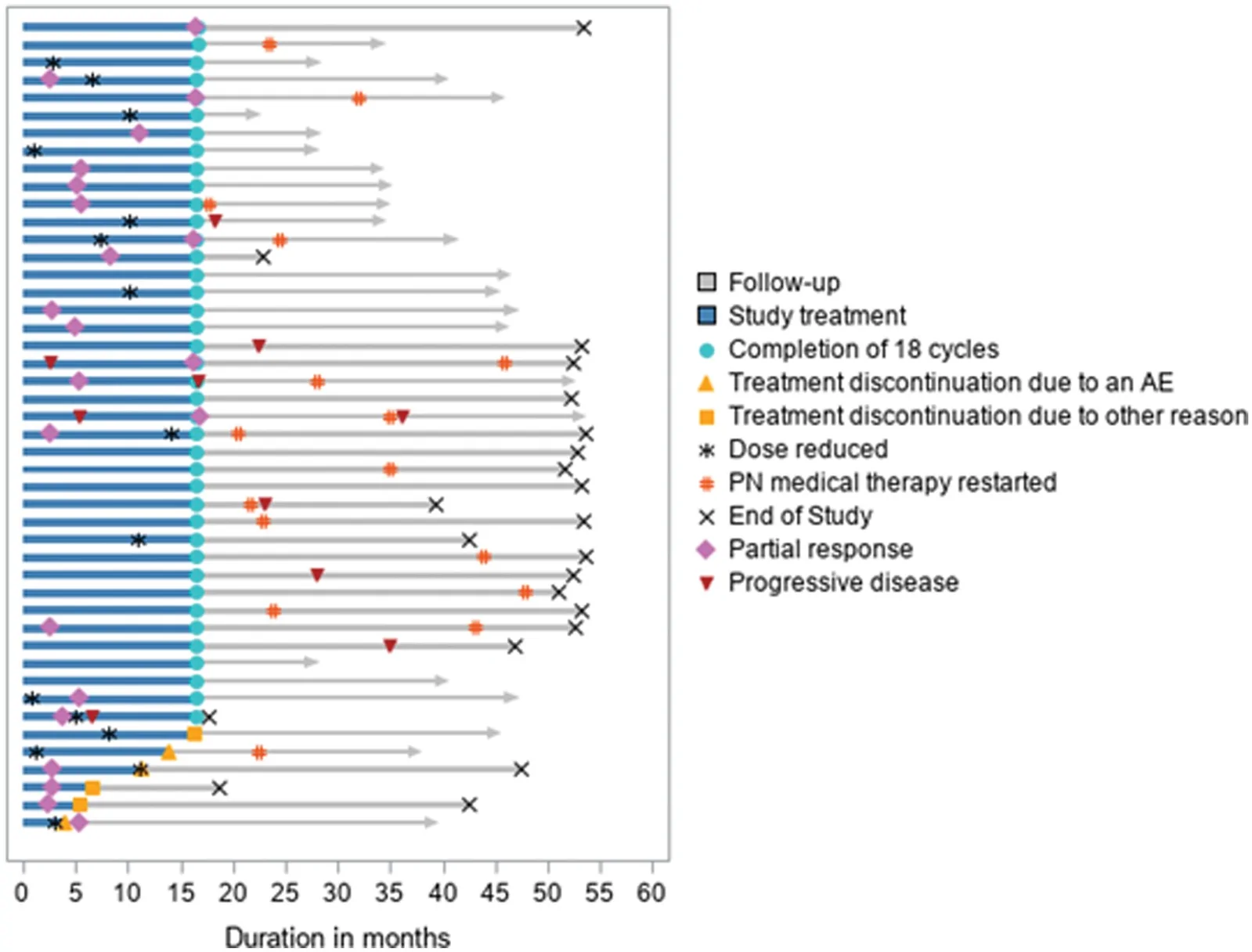
Swimmers plot of patients with NF1 and plexiform neurofibromas. Duration of treatment and response (volume compared to baseline).

When using volumetric evaluation, 5 patients (10.9%) had progression at some point during treatment when compared to baseline (Figure 3 and Figure SA2) and one more patient (2.2%) had a progression during treatment when compared to the smallest volume from previous timepoints. Three patients with volumetric progression compared to baseline continued treatment since no progression was identified on RECIST 1.1 evaluation by local investigator and there was no clinical progression. Two had subsequent partial response at the final assessment of the end of treatment (18 cycles).

After discontinuation of treatment, 5 (10.9%) patients had volumetric progression when compared to baseline. Compared with the smallest volume recorded, 13 (28.2%) patients developed progressive disease after treatment discontinuation.

When compared to baseline, progression-free survival (PFS) for the volumetric evaluation was 73.1% (95% CI 53.3-85.5) at 36 months and the median PFS was not reached (Figure SA3). When compared to the smallest volume during treatment, the PFS was 57.0% (95% CI 38.1-72.0) at 36 months and the median PFS was 40.3 months (95% CI 23.2-not estimable) (Figure SA2).

**Figure 4.**
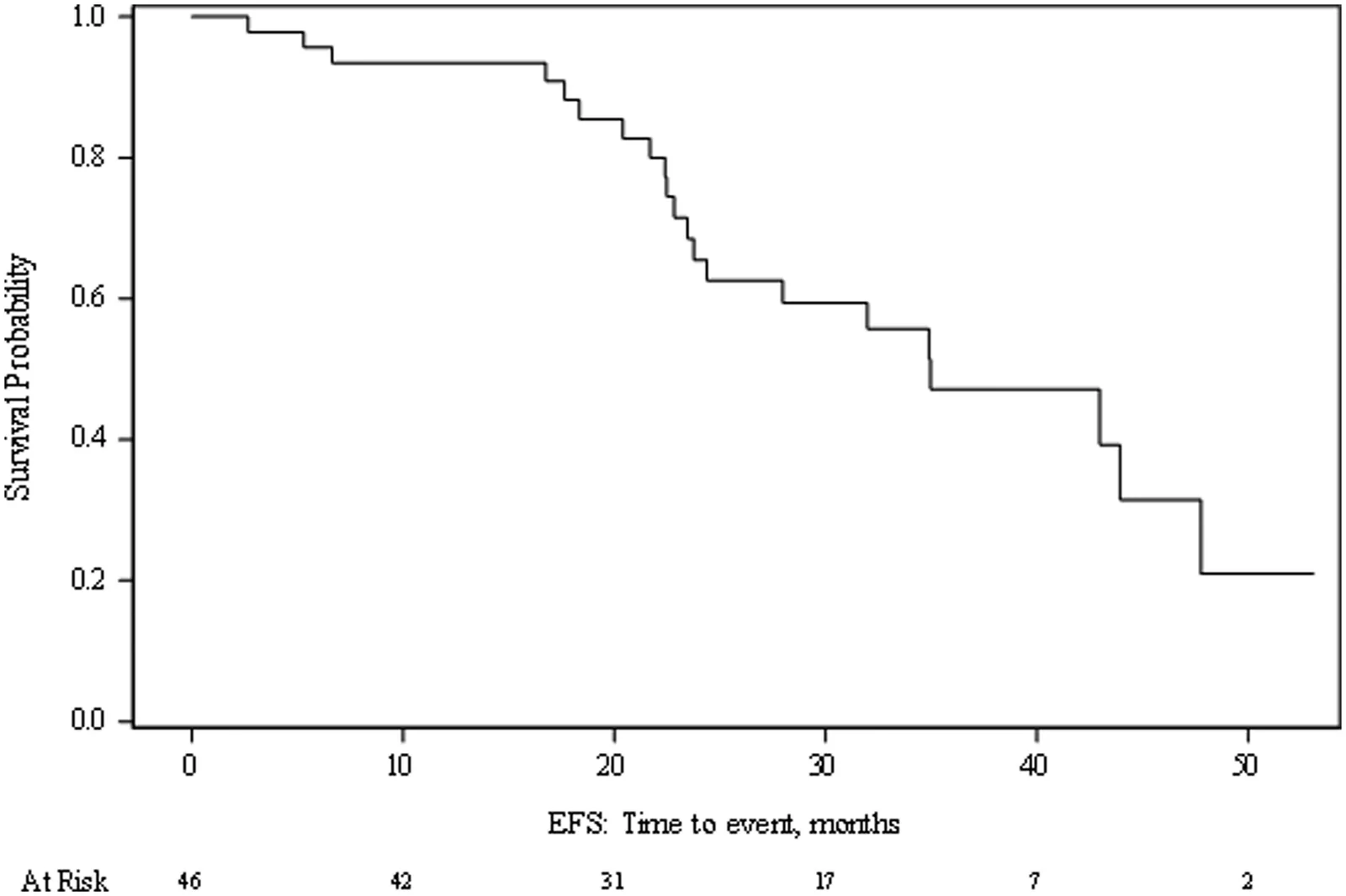
Event-free survival of patients with NF1 and plexiform neurofibromas from treatment initiation to an event defined as either a progression of the lesion based on volumetric assessment compared to baseline or any intervention in the intend to treat the PN following discontinuation of treatment.

The 3 years EFS was 47.1 % (95% CI 28.8-63.5) and the median EFS was 35 months (95% CI 23.8-47.8) (Figure 4). At time of data cut off, a total of 15 (32.6%) patients had interventions intended to treat the PN following discontinuation of treatment. Eleven (23.9%) patients restarted a MEKi and 4 (8.7%) underwent surgery. The median time to restart treatment after discontinuation was 10 months (range 1.1-31.2 months). Reasons reported by local investigators for restarting treatment included disease progression (*n* = 10), pain (*n* = 6), and visual impact (*n* = 1). Four patients experienced a combination of progression and pain. One patient with progressive optic pathway glioma was started on weekly vinblastine.

One patient without tumor shrinkage had pain that responded rapidly to trametinib. The patient had slow growth during treatment and pain restarted rapidly at the end of treatment. She was later found to have a malignant peripheral nerve sheath tumor 22 months after enrolment.

### Toxicity

Most AEs were grade 1 or 2 and included predominantly skin and gastro-intestinal toxicities (Table SA3). Fifteen patients (32.6%) had a dose reduction due to AEs and most were related to cutaneous toxicity (4 acneiform rash, 3 paronychia and 2 mucositis; Table SA4). Even though dose reduction, 6 (40%) achieved a volumetric response during treatment which was not significantly different than patients without dose reduction (51.6% *P* = .5). Three patients (6.5%) discontinued treatment due to AEs (paronychia, generalized oedema and pneumonitis). Two patients (4.3%) refused further treatment (at cycle 6 and 8, respectively).

### Patient-Reported Outcome Measures

Analyses of patient reports revealed significant changes in overall quality of life scores over time (brain tumor module, *P* = .043 and generic core scales, *P* = .015; Table 2). Specifically, children reported significantly better cognitive scores (mean diff: 13.67; SE 3.89; *P-*value = .01; 95% CI 2.57-24.77) and school functioning (mean diff: 10.39; SE 3.28; *P-*value = .022; 95% CI 1.06-19.71) at cycle 13 compared with cycle 7. No significant changes were observed between baseline and the end of the treatment for either patient reports and parent-proxy reports. Parent’s perception of their child’s quality of life remained stable over time, expect for increased worry at cycle 7 (mean diff: −14.05; SE 4.49; *P-*value = .021; 95% CI −26.62 to −1.48) and cycle 13 (mean diff: −10.00; SE 3.15; *P-*value = .019; 95% CI −18.81 to −1.19), compared with baseline (Table SA5).

**Table 2.** Descriptive analyses of PedsQL brain tumor module, generic core scales for child self-report by subscale.

	Baseline	Cycle 7	Cycle 13	End of treatments	*F*	*P*-value	η*p* ^2^
	Mean (SD)	Mean (SD)	Mean (SD)	Mean (SD)			
PedsQL brain tumor module (*N* = 27)							
Cognitive problems	64.95 (19.71)	64.20 (16.66)	77.87 (20.15)	67.86 (21.16)	4.906	.008	0.38
Pain and hurt	69.75 (20.43)	74.07 (14.12)	82.10 (18.66)	75.77 (20.67)	3.209	.041	0.286
Movement and balance	83.95 (22.04)	89.81 (13.93)	89.51 (17.69)	88.58 (18.51)	0.615	.612	0.071
Procedural anxiety	59.26 (30.69)	74.07 (27.28)	75.93 (31.46)	71.30 (32.22)	3.003	.05	0.273
Nausea	90.19 (13.48)	88.61 (14.43)	91.85 (9.11)	87.50 (12.69)	2.198	.114	0.216
Worry	71.14 (22.97)	69.91 (25.85)	83.64 (17.83)	79.01 (18.40)	2.731	.066	0.254
Overall score	73.21 (14.96)	76.78 (13.73)	83.48 (14.68)	78.34 (15.07)	3.151	.043	0.283
PedsQL generic core scales (*N* = 29)							
Physical functioning	72.58 (20.13)	74.25 (18.61)	77.03 (22.26)	78.13 (20.93)	1.533	.229	0.150
Emotional functioning	70.17 (18.00)	70.69 (20.30)	73.28 (18.52)	74.18 (16.22)	0.993	.412	0.103
Social functioning	72.59 (19.28)	77.13 (16.19)	82.89 (18.38)	79.83 (18.64)	2.908	.054	0.251
School functioning	63.97 (18.44)	62.37 (17.94)	72.76 (14.86)	63.19 (25.44)	3.546	.028	0.290
Overall score	69.82 (15.18)	71.11 (14.18)	76.49 (15.38)	73.83 (15.86)	4.225	.015	0.328

To note, end of treatment PedsQL^TM^ assessments were obtained after discontinuation of trametinib in 33 patients. PedsQL^TM^ were completed at a median of two days after end of treatment (range—7 to 43 days).

## Discussion

We report the results of the first large cohort of patients with NF1 and PN treated prospectively on study with trametinib. We demonstrate the efficacy and tolerance of trametinib for the treatment of PN. This study helps understand the evolution of PNs once MEKi are discontinued.

Using the PN volumetric assessment, we demonstrated an ORR of 47.8% with a median volume change of −19.3%. Our results are similar to those reported with mirdametinib (52%) but lower than the ORR reported with selumetinib (68%) and binimetinib (74%) (2-5). There are several limitations when comparing the efficacy of different MEK inhibitors across studies, including variations in patient selection, treatment duration and methods used to assess volumetric responses. In our study, patients were treated for a maximum of 18 cycles, whereas patients included in the phase 1/2 selumetinib studies could continue indefinitely if there was evidence of clinical benefit.^2^ It is possible that with a longer duration of treatment more patients could have achieved a partial response. The median time to response in our study was 5.3 months but four patients had their best responses at their final evaluation prior to discontinuation. The median time to best response was 16 cycles (range 4-36 cycles) in the selumetinib study and 13.4 months (range, 4.0-32.7) in the mirdametinib study.^2^^,^^3^ Another notable difference was the tumor location and size. While the median volume was 487 ml (range: 5-3820) in the selumetinib studies, our patients had much smaller PN (median 104.5 ml). This could be explained by the fact that more than a third of our patients had PNs involving the head, which are typically smaller. Our population was more similar to those in the mirdametinib pediatric group where median PN volume was 99 ml and 50% of PN involved the head and neck.^3^

Overall, treatment was well tolerated with mainly grade 1 and 2 AEs. Most AEs were associated with skin reaction and localized infection such as paronychia. Less than a third of patients required a dose reduction and 3 discontinued treatment. These percentages are similar to those reported in selumetinib, binimetinib and mirdametinib studies (12% to 42% dose reduction).^2^^,^^3^^,^^6^ However, comparison is limited due to the different durations of treatment. Similar types of AEs were also reported in these clinical trials.

We did not observe significant association between tumor location, tumor volume, age or trametinib serum level and the rate of tumor response. Patients with distinct nodular lesions appeared to have trend toward lower response rate but larger studies or pooled analysis from combined studies are needed to confirm this observation.^17^

Consistent with recommendations to integrate PROs in clinical trials,^18^^,^^19^ patients’ quality of life was assessed at baseline and during treatment. Children reported overall improvements in quality of life over time, with significant improvements in cognitive scores and school functioning. In contrast, the parents’ overall perception of their child’s quality of life remained stable, although they reported greater worry at cycles 7 and 13 compared with baseline. Interestingly, most subscales showed a trend toward improvement at cycle 13 followed by deterioration at the end of treatment. This may be explained by the fact that many questionnaires were completed after trametinib discontinuation. This pattern suggests that improvements observed at cycles 7 and 13 could be associated with ongoing treatment. Pain and hurt scores also changed over time (*P* = .041), but post hoc analyses did not identify where differences occurred, likely due to the conservative Bonferroni adjustment. Unlike other clinical trials, no specific pain assessment was included in our study. While patients likely benefited from pain reduction at treatment initiation, this effect could not be fully captured. Overall, our findings indicate that treatment, adverse events, clinic visits, and examinations did not negatively affect quality of life. These results also align with our recent study demonstrating improvements in standardized cognitive measures of executive function.^20^

The optimal duration of treatment with MEKi for PN remains unknown. Since all patients on our study discontinued treatment after a maximum of 18 cycles, our clinical trial offers a unique window to better understand the evolution of PN at discontinuation. Even though most patients had tumor shrinkage during treatment almost a third of patients (32.6%) required a new intervention following discontinuation. The median EFS was 35 months and the median time to restart treatment after discontinuation was 10 months. Most of these patients did not meet criteria for progressive disease by volumetric evaluation but patients, families and providers decided to proceed with a new intervention to better control their PN or address symptoms. As our cohort continues to mature and patients complete the follow-up period, we will be able to better appreciate patient outcomes and the efficacy of MEKi once restarted. Clinical characteristics associated with prolonged stability after discontinuation is still unknown but some information might be provided with the long-term follow-up of our cohort and other studies.

Patients with progressive plexiform neurofibromas, associated clinical impact, or impending morbidity were eligible. Although only 4 patients demonstrated volumetric progression (≥20% increase) prior to enrollment, 31 were identified by local investigators as having progressive neurofibromas based on an observed increase in size. Our ability to confirm volumetric progression was limited: only a single pre-baseline MRI was mandated by the study protocol, the timing of this scan was not standardized, and some clinically obtained MRIs lacked the sequences necessary for volumetric assessment. Consequently, only 4 patients had confirmed volumetric progression at enrollment.

We believe this limitation does not meaningfully affect the clinical relevance of our findings. In the SPRINT trial, 42% of participants had progressive plexiform neurofibromas at baseline, compared with 62% in the ReNeu study, and neither study demonstrated meaningful differences in response rates between patients with progressive versus non-progressive disease at baseline.^2^,3 Nonetheless, we acknowledge that this question warrants further study and emphasize the importance of systematically collecting several pre-baseline MRI scans to better characterize PN growth trajectories before and after treatment.

One limitation of our study is that real-time evaluation of the volumetric response was not possible and tumor responses were based on local investigators using RECIST 1.1 criteria. Five patients were retrospectively found to have progressed during treatment compared to baseline and would have been removed from treatment if evaluations had been based on volumetric response. Despite this, 3 out of 5 patients responded later during treatment suggesting that some patients might have progressed before responding to MEKi.

The importance of volumetric assessment is also demonstrated by the high discordance between volumetric analysis and one-dimension measurements. In 41.3%, the response was either under or overestimated with one-dimensional assessment.

Another limitation of our study is that specific assessment of pain and function outcome was not included. It will be critical for new upcoming studies to integrate these evaluations as suggested by the Response Evaluation in Neurofibromatosis and Schwannomatosis (REiNS).^21^

## Conclusion

Our study demonstrates that trametinib is an effective treatment for PN and is usually well tolerated. The efficacy of trametinib is maintained during treatment but a significant percentage of patients need to restart treatment after discontinuation. Volumetric assessment of PN is essential to accurately evaluate tumor bulk. The evolution of patients in the follow-up phase will continue to provide information regarding the outcome of patients treated with trametinib.

## Supplementary Material

noag112_Supplementary_Data

## Data Availability

Patient data are not publicly available to protect patient privacy but is available upon reasonable request to the corresponding author (Sébastien Perreault, s.perreault@umontreal.ca). Provided data will be de-identified.
