# Supplementary material for: TRAM-01: A phase 2 study of trametinib for pediatric patients with neurofibromatosis type 1 and plexiform neurofibromas
Source: Neuro Oncol. 2026 May 19;28(9):2279–88. doi: 10.1093/neuonc/noag112 (PMC13550690; doi:10.1093/neuonc/noag112)
Supplement: Supplementary file 1 [file noag112_supplementary_data.docx]

**Appendices**

**Table A1: Eligibility and exclusion criteria**

| Study Inclusion Criteria | |
| --- | --- |
| 1. Signed written informed consent | Prior to study participation, written informed consent from participants, or in the case of minors, written permission (informed consent) from parents, guardians, or legally acceptable representatives must be obtained according to local laws and regulations. |
| 2. Assent | Assent from minor participants should be obtained per local laws and regulations and should be documented in accordance with local requirements. |
| 3. Study activities compliance | Participants must be willing and able to comply with scheduled visits, treatment schedule, laboratory testing, and other requirements of the study, including disease assessment by contrast enhanced MRI |
| 4. Age | Patients must be aged ≥ 1 month (corrected age) to ≤ 25 years when starting trametinib. |
| 5. Study group | Participants must belong to one of the following groups to be eligible.  • Group 1: NF1 with Progressing/Refractory LGG (42 patients).  • Group 2: NF1 with PN (46 patients).  • Group 3: Progressing/Refractory LGG with KIAA1549- BRAF fusion (42 patients).  • Group 4: Progressing/Refractory Glioma with activation of the MAPK/ERK pathway who do not meet criteria of other study groups (20 patients). |
| 6. Tumor Tissue Sample | Tumor tissue will be required for all patients (minimally paraffin- embedded and additionally fresh frozen tissue [if available]). Patients with NF1 and LGG or PN can still be enrolled without tissue if no surgery or biopsy was conducted. |
| 7. Previous MRI | At least two previous MRIs for Group 1,3,4 and one previous MRI for Group 2 must be available for central review. |
| 8. Prior therapy | Participants must have failed at least one line of treatment including chemotherapy and/or radiation therapy except for plexiform neurofibroma (PN) (since there is no recognized standard treatments for this tumor). |
| 9. Prior therapy toxicity | Patients must have recovered to grade ≤ 1 from acute toxic effects of all prior chemotherapy, immunotherapy or radiotherapy prior to enrollment except for alopecia and non-treatment related clinically insignificant laboratory abnormalities prior to starting trametinib. Toxicities will be graded as per the National Cancer Institute Common Terminology Criteria for Adverse Events (CTCAE), version 5·0. |
| 10. Prior therapy timeline | Participants having previously received a chemotherapy agent(s) and/or radiation must conform to the timeline described below. There is no limitation on the number of previous treatments or cycles received.  An interval of at least 28 days after the last dose of a myelosuppressive chemotherapy, and at least 42 days after the last dose of Nitrosoureas  is required prior to starting trametinib.  An interval of at least 28 days after the last dose of biologic agents including monoclonal antibody treatment, immunotherapy, viral therapy and other investigational agent is required prior to starting trametinib.  An interval of at least 84 days after the end of the radiation therapy is required prior to starting trametinib.  An interval of at least 48 hours for short acting colony stimulating factor agents, and 10 days interval for long acting colony stimulating factor agents are required prior to starting trametinib. |
| 11. Life expectancy | Patients must have a life expectancy of greater than 6 months. |
| 12. Performance level | Patients must have a performance status corresponding of a Lansky/Karnofsky score ≥50. |
| 13. Organ Function Requirements | Participants must have normal organ and marrow function as defined below:  • Total leukocytes ≥ 3,000/μL (3*109/L)  • Absolute neutrophil count (ANC) ≥ 1, 000/μL (1*109/L)  • Hemoglobin > 80 g/l (transfusion independent within last 2 weeks prior to starting trametinib)  • Platelet count ≥ 100,000/μL (100*109/L) (transfusion independent within last 2 weeks prior to starting trametinib)  • Total bilirubin ≤ 1·5X ULN within normal institutional limits for age (patients with documented Gilbert’s Disease may be enrolled with Sponsor approval and total bilirubin ≤ 2·0 × ULN)  • Alanine Aminotransferase (ALT) ≤ 2·5 × upper limit of normal  (ULN)*  • Creatinine serum under or within normal institutional limits for age OR  creatinine clearance ≥60 mL/min/1·73 m2 for participants with creatinine levels above institutional normal.  • Creatine phosphokinase ≤ 2x ULN  • Cardiac function defined as Corrected QT (QTcB) interval < 480 msec and LVEF ≥ lower limit of normal (LLN) by ECHO.  • Blood pressure must be smaller or equal to the 95th percentile for patient’s age, height and gender.  * For uniformity reasons, the ULN for ALT will be 45 U/L in this study. |
| 14. Reproductive status | Female and male patients of fertile age must agree to use adequate contraception (hormonal or barrier method of birth control; abstinence) prior to starting trametinib, for the duration of study participation and for 6 months after the completion of trametinib administration. Female patients of childbearing potential (≥ 10 years old for this study) must have a negative serum pregnancy test within 7 days prior to starting trametinib. |
| 15. Administration of oral medication | Patients must be able to ingest and retain enterally (per os, nasogastric tube or gastrostomy) administered medication and be free of any clinically significant gastrointestinal abnormalities limiting the absorption of medication. Tablets cannot be crushed. If the patient cannot swallow tablets, the liquid form should then be used. |
| Study Exclusion Criteria | |
| 1. Other investigational agents | Patients who are receiving any other investigational agents. |
| 2. Cardiac exclusion criteria | Patients who have an ejection fraction inferior to the institution LLN, a QTcB ≥ 480 msec or an absolute resting left ventricular ejection fraction (LVEF) of ≤ 39% are not eligible for enrollment. |
| 3. Presence of another malignancy | Patient has any other malignancy except if the other primary malignancy is neither currently clinically significant nor requiring active intervention. |
| 4. Previous MEK inhibitor treatment | Participants previously treated with a MEK inhibitor who showed less than stable disease during treatment. |
| 5. BRAF V600E mutation | Patients with a tumor presenting a BRAF V600E mutation. |
| 6. Other uncontrollable medical disease | Patient has a severe and uncontrollable medical disease (i.e.,  uncontrolled diabetes, chronic renal disease or active uncontrolled infection), has chronic liver disease (i.e., chronic active hepatitis and cirrhosis), uncontrolled intercurrent illness including, but not limited to, ongoing or active infection, symptomatic congestive heart failure, unstable angina pectoris, cardiac arrhythmia, or psychiatric illness/social situations that would limit compliance with study requirements. |
| 7. Known HIV infection | Patient has a known diagnosis of human immunodeficiency virus (HIV) infection, hepatitis B or C. |
| 8. Previous surgery | Patients who had major surgery within 2 weeks prior to starting trametinib. |
| 9. Allergy | History of allergic reactions attributed to compounds of similar chemical or biologic composition to trametinib. |
| 10. Previous history of non-compliance | Patients with previous significant history of non-compliance to their treatment or medical regimen. |
| 11. Pregnant or breastfeeding patients | Pregnant or breastfeeding female patients are not eligible for this study. |

**Data A1: Dose modification and toxicity management**

The descriptions and grading scales found in the revised CTCAE version 5·0 will be utilized for AE reporting. All appropriate treatment areas should have access to a copy of the CTCAE version 5·0.

| OPHTHALMOLOGIC TOXICITY | | | |
| --- | --- | --- | --- |
|  | **Event Grade** | **Description of grade / Management** | **Treatment Dose Modification and Actions** |
| Management of Visual Changes and stopping criteria  RPED: Retinal Pigment Epithelial Detachments.  RVO: Retinal Vein Occlusion. | 1 | Within the first 7 days of onset, consult with an ophthalmologist. | Continue treatment at the same dose following the exclusion of RPED or RVO. Diagnosis or suspicion of RPED: Follow the specific guidelines detailed in Table 9. Diagnosis of RVO: Discontinue treatment immediately and permanently. Report RVO as an SAE.  Diagnosis of uveitis: Follow the specific guidelines detailed below |
|  | 2-3 | Immediately consult with an ophthalmologist. | Continue treatment at the same dose following the exclusion of RPED or RVO. Diagnosis of RPED: Follow the specific guidelines detailed below. Diagnosis of RVO: Discontinue treatment immediately and permanently treatment. Report RVO as an SAE.  Diagnosis of uveitis: Follow the specific guidelines detailed below |
|  | 4 | Immediately consult with an ophthalmologist. | Exclusion of RPED and RVO: hold treatment and discuss the event with PI of Study to assess the situation and consider treatment restart. Diagnosis of RPED or RVO: Discontinue treatment immediately and permanently. Report as an SAE.  Diagnosis of uveitis: Follow the specific guidelines detailed below |
| Management of retinal pigment epithelial detachments (RPED) and stopping criteria | 1 | Asymptomatic | Maintain study treatment and implement a monthly evaluation until resolution. If there is worsening of RPED, follow the management criteria for Grade 2-3 RPED. |
|  | 2-3 | Mild to moderate decrease in visual acuity. Symptomatic | Hold trametinib treatment. Follow participant with a retinal evaluation within 3 weeks. Upon resolution to baseline or grade 1, trametinib may be resumed at a reduced dose. If RPED does not improve to at least Grade 1 within 3 weeks OR recurrence of RPED (any Grade) after dose interruption or reduction, study treatment should be permanently discontinued. |
|  | 4 |  | Refer to management of visual changes and stopping criteria |
| Management of uveitis | Any | - | Maintain study treatment and implement ocular therapy. If no improvement within 2 weeks hold treatment until adverse reaction resolves and resume at same dose. If recurrence of uveitis (any Grade) reduce dose. |

| CARDIAC TOXICITY | | | |
| --- | --- | --- | --- |
|  | **Event Grade** | **Stopping criteria** | **Treatment Dose Modification and Actions** |
| QTc Stopping Criteria | - | For the QTcB (Bazett’s formula) stopping criteria and follow-up herewith described, the prolongation must be observed on at least 3 different ECGs over one week period.  If QTcB is ≥ 500 msec, take the following actions:  Perform a review of concomitant medication known to prolong QTc.  Obtain and document serum levels of magnesium, phosphorus, potassium and calcium.  Hold study treatment and wait until resolution to baseline or grade 1.  If there is no recovery to baseline in the 4 weeks following the event, discontinue permanently study.  Following resolution, you may restart study medication at the same dose level. To resume the treatment, it must be determined that the patient could benefit from further treatment after discussion between the Sponsor-Investigator and treating Investigator. If there is any re-occurrence of a QTcB of ≥ 500 msec, discontinue study treatment permanently. | |
| Stopping Criteria and Treatment Dose Modification for decrease of LVEF  * Recovery definition: absolute decrease of 10% or less compared to baseline and LVEF ≥ LLN.  ** All follow-up ECHO should be done within ±3 days from the targeted date. | 2 | Absolute LVEF decrease from baseline of 10% to 19% and ejection fraction below the institution’s LLN or Resting LVEF of 50 - 40%; | Hold treatment immediately.  Within 2 weeks of the observation, repeat ECHO**. If no resolution of LVEF on subsequent ECHO, repeat ECHO 2** weeks after the first one. Consult with a cardiologist. In the event of LVEF recovery* in the 4 following weeks: Contact PI of Study to discuss possible restart of patient’s treatment. Repeat ECHO** at week 2, 4, 8 and 12 following the restart of treatment. If no decrease of LVEF, continue ECHO** at intervals of 12 weeks.  If no recovery from LVEF in the following 4 weeks. Study treatment should be **permanently** discontinued. Repeat ECHO** at week 2, 4, 8, 12 and 16 following event or until resolution. Report the event (SAE) as per the procedures in section 6·2 |
|  | 3 | Absolute LVEF decrease from baseline of 20% or more and ejection fraction below the institution’s LLN or Resting LVEF of 39-20% | Discontinue Trametinib **permanently**. Repeat ECHO** at week 2, 4, 8, 12 and 16 following event or until resolution. Report the event as an SAE. |
|  | 4 | Resting LVEF of less than 20%. |  |
| Management of Cardiac Valvular Toxicity  *Mitral/tricuspid/aortic valvular toxicity as per NCI-CTCAE. ** All follow-up ECHO should be done within ± 3 days from the targeted date. | 2-3 | Presence of new asymptomatic, moderate regurgitation or stenosis by ECHO*. | Hold study treatment temporarily. Repeat ECHO within two weeks** of the first observation and every 2 weeks** until recovery to baseline or until 4 weeks has elapsed. In the event of baseline recovery of the valve in the 4 weeks following first observation, contact PI of study to consider restart of study. Following restart, perform ECHO as per the following schedule: Repeat ECHO** at week 2, 4, 8 and 12 following the restart of treatment.  If no valve dysfunction, continue ECHO at intervals of 12 weeks. If no valve recovery to baseline in the following 4 weeks.  Study treatment should be **permanently** discontinued.  Repeat ECHO** at week 2, 4, 8, 12 and 16 following event or until resolution. |
|  | 4 | Presence of symptomatic, severe regurgitation/stenosis by ECHO, with medical intervention for the control of symptoms. | Hold study treatment immediately.  Repeat ECHO** within one week of the first observation and every 1-2 weeks until recovery to baseline or 4 weeks If resolution to baseline on ECHO and of symptoms occurs within 4 weeks of event, contact PI of Study to consider restart of study treatment at a reduced dose.  If no valve recovery to baseline in the following 4 weeks.  Study treatment should be **permanently** discontinued. Repeat ECHO** at week 2, 4, 8, 12 and 16 following event or until resolution. |

| HYPERTENSION | |
| --- | --- |
| Blood Pressure (BP) value | **Treatment Dose Modification and Actions** |
| BP ≤ 95^th^ percentile^a^. | Continue study treatment at the same dosage. |
| BP ≤ 10 mmHg above the 95^th^ percentile. | Continue study treatment at the same dosage. BP should be measured at least twice during the following week. If BP is smaller or equal to the 95^th^ at re-evaluation, continue study treatment without dose modification. If BP is still above the 95^th^ at re-evaluation, then start/adjust antihypertensive therapy. |
| BP is ≥ 11 to 25 mm Hg above the 95^th^ percentile on ≥ 2 of 3 measurements or > 35 mmHg above baseline on ≥ 2 of 3 measurements. | Start/adjust anti-hypertensive therapy and continue trametinib at the same dose. Monitor BP at least twice weekly.  If the BP returns to ≤ 95^th^ within 14 days, continue trametinib at the same dose and continue anti-hypertensive therapy. If the BP remains ≥ 11 to 25 mm Hg above the 95^th^ or > 35 mm Hg above baseline for more than 14 days after the institution/adjustment of anti-hypertensive therapy, hold trametinib, monitor BP at least every 3 days. The anti- hypertensive therapy should be continued until the BP is less than the 95^th^.  If the BP returns to ≤ 95^th^ within 14 days (28 days of first reported event), restart trametinib at a reduced dose. If the BP remains ≥11 to 25 mm Hg above the 95^th^ or > 35 mm Hg above baseline despite optimal anti-hypertensive therapy and after 14 days (28 days of first reported event) without trametinib, discontinue study treatment permanently. |
| If BP is > 25 mm Hg above the 95^th^ percentile. | Hold trametinib, monitor BP at least twice weekly, and administer/adjust anti-hypertensive therapy as clinically indicated. If the BP returns to ≤ 95^th^ within 14 days, trametinib may be restarted at a reduced dose. If the BP is > 25 mm Hg above 95^th^ for >14 days, discontinue study treatment permanently.  If the participant develops Grade 4 hypertension, discontinue trametinib permanently, monitor BP and administer anti-hypertensive therapy as clinically indicated. |

^a^For patient age, height and gender.

| LIVER TOXICITY | |
| --- | --- |
| Liver Chemistry Stopping Criteria | **Follow-up procedures** |
| ALT ≥ 3x the ULN and total bilirubin ≥ 2x the ULN umol/l or  ALT ≥ 3x the ULN and international normalization ratio (INR) ≥ 1·5. | Subject must come back to site in the 24 hours following the event for further testing, liver chemistries, additional tests and monitoring.  If possible, please consult with an hepatologist.  Patient must be monitored twice weekly until the following liver chemistry resolve, return to baseline values or stabilize: Alanine Aminotransferase (ALT), Gamma Glutamyl Transferase (GGT), Aspartate Aminotransferase (AST), Alkaline Phosphatase (ALP), and Total bilirubin |
| For all patients who are meeting a liver chemistry stopping criteria, all possible efforts must be made to carry the following testing procedures: | • Perform viral hepatitis serology:   IgM antibody for Hepatitis A   Hepatitis B IgM as well as Hepatitis B surface antigen   Epstein-Barr viral capsid antigen IgM antibody   Hepatitis C RNA   Cytomegalovirus IgM antibody  • Complete Blood Count (CBC) with differential  • If ALT ≥ 3x the ULN perform INR  • If total bilirubin is ≥ 2x the ULN, perform conjugate bilirubin  • Lactate Dehydrogenase (LDH) and Serum Creatinine Phosphokinase (CPK)  • All clinical symptoms associated with hepatitis or hypersensitivity reaction, such as fatigue, nausea must be recorded as per section 6·2  • Record all concomitant medication including any over-the-counter (OTC) medication (Ex: Acetaminophen and herbal remedies) • Record patient’s use of alcohol  • For subjects with ALT ≥ 3x ULN and total bilirubin ≥ 2x the ULN:   Liver imaging for the evaluation of liver disease (MRI, CT-Scan or Ultrasound)   Type 1 anti-liver kidney microsomal antibody, anti-smooth muscle antibody, anti- nuclear antibody |

| SKIN REACTIONS | | | |
| --- | --- | --- | --- |
|  | **Event Grade** | **Management** | **Treatment Dose Modification and Actions** |
| Management in case of Rash Acneiform | 1 | Initiate the prophylactic treatment measures. Reassessment must be done after **2 weeks**. | Trametinib treatment can be continued. In the event the rash does not return to baseline in the 2 weeks following the implementation of topical agents (mild topical steroid and topical antibiotics), consider use of doxycycline or minocycline. If there is no improvement, consider use of isotretinoin. |
|  | 2 | Initiate the prophylactic and symptomatic treatment. Reassessment must be done after **2 weeks**. | Continue trametinib.  Begin medium potency topical steroid treatment.  Optimize topical antibiotics and oral antibiotics.  Consider use of isotretinoin.  If there is no improvement of the rash within four weeks, reduce dose.  At the 2-week reassessment after dose reduction: Continue with trametinib at reduced dose if the event resolved to baseline or grade 1. If no resolution of the event to baseline or grade 1, hold trametinib treatment for a maximum of 4 weeks until resolution to grade 1 or less. Trametinib may be resumed at a reduced dose following resolution. If not recovered to Grade 1 or less in 4 weeks, then discontinue trametinib permanently. |
|  | 3-4 | Consult a Dermatologist | Immediately hold trametinib treatment until event resolves to baseline or grade ≤ 2. Following resolution, resume treatment at a reduce dose and follow grade 2 or grade 1 Rash management, as applicable.  In the event the rash has not resolved to grade ≤ 2 or less in 4 weeks, **discontinue trametinib permanently**. |

| PNEUMONITIS AND INTERSTITIAL LUNG DISEASE | | |
| --- | --- | --- |
|  | **Event Grade** | **Treatment Dose Modification and Actions** |
| Management of Pneumonitis and Interstitial lung disease | Any | Permanently discontinue study medication. |

| DIARRHEA | | |
| --- | --- | --- |
|  | **Event Grade** | **Treatment Dose Modification and Actions** |
| Management of Diarrhea related to trametinib^a^  ^a^ Symptoms must be ongoing for more than 72h and related to trametinib ^b^ Loperamide is not recommended for children ˂ 2 years. | 1-2 | Modify patient’s diet: consume smaller meals and stop all lactose containing products. The implementation of a simple diet such as banana, rice, apple and toast can be considered. Patient’s oral hydration should be encouraged  If patient is 2 years or older^b^, take into consideration the administration of loperamide. Loperamide can be discontinued if patient is free of diarrhea for at least 12 hours.  If patient’s diarrhea resolves to baseline or grade 1 in the 72h following the implementation of management criteria cited above, study treatment may be continued without dose modification.  If patient’s diarrhea remains Grade 2 for 72h following the implementation of management criteria, hold trametinib treatment. If within 7 days diarrhea: Resolves to Grade 1 or baseline: resume trametinib at full dose Does not resolve to ˂ Grade 2, discontinue study treatment permanently.  If the patient’s diarrhea recurs (Grade ≥2 for 72 h) despite optimal management when patients resume trametinib discontinue study treatment permanently. |
|  | 3-4 | Hold trametinib for a maximum of 7 days until the event resolves to baseline or Grade 1. Refer to Grade 1-2 for management. For uncontrolled diarrhea with severe dehydration consider the administration of octreotide. If event does not resolve to < Grade 2 within 7 days, discontinue study treatment permanently |

| FEBRILE DRUG REACTION | | |
| --- | --- | --- |
| Description | **Treatment Dose Modification and Actions** | |
| If fever of 38·5 – 40 °C without complications | | Continue study treatment at same dose. |
| If fever > 40 °C or any fever with complications due to rigors, hypotension, dehydration or renal failure. | | Hold study treatment until resolved and resume at the same dose. |

| GENERAL DOSE MODIFICATION CRITERIA FOR OTHER EVENTS CONSIDERED RELATED TO TRAMETINIB | | |
| --- | --- | --- |
|  | **Event Grade** | **Treatment Dose Modification and Actions** |
| Management of Other Toxicities Related to Trametinib | 1 or 2 (tolerable) | Maintain treatment at the same dose level. To assess resolution or deterioration, closely monitor patient. As clinically indicated, provide supportive care as per your institutional guidelines. |
|  | 2 (intolerable) or 3 | Hold trametinib administration. To assess resolution or deterioration, closely monitor patient. As clinically indicated, provide supportive care as per your institutional guidelines. If resolution to baseline or Grade 1 occurs within 4 weeks, you may resume trametinib administration with a reduced dose. If the same Grade 2 (intolerable) or 3 event reoccurs OR does not resolve to Grade 1 or baseline in 4 weeks; permanently discontinue study medication. |
|  | 4 | Hold trametinib administration. To assess resolution or deterioration, closely monitor patient.  As clinically indicated, provide supportive care as per your institutional guidelines. If event resolves within 4 weeks to baseline or grade 1: Contact PI of Study to discuss patient’s continuation in study. If continuation is possible, resume treatment at a reduced dose. If there is no resolution of adverse event within 4 weeks, permanently discontinue study medication. If the same Grade 4 event reoccurs OR does not resolve to Grade 1 or baseline in 4 weeks; permanently discontinue study medication. |

**Data A2:**

**PATIENT-REPORTED OUTCOME MEASURES**

The PedsQL brain tumor module, validated in English(9) and French(10), is composed of 24 items multidimensionally distributed on six scales: (1) cognitive problems (7-items), (2) pain and hurt (3-items), (3) movement and balance (3-items), (4) procedural anxiety (3-items), (5) nausea (5-items), and (6) worry (3-items). This questionnaire has excellent psychometric properties with Cronbach's alpha coefficients for the total scale score of an average α = 0.76-0.98 for child self‐report and an average α = 0.78-0.98 for parent proxy-reports(9, 10).

The PedsQL generic core scales, validated in English (7) and French (11), are composed of 23 items multidimensionally distributed on four scales: (1) physical functioning (8-items), (2) emotional functioning (5-items), (3) social functioning (5-items), and (4) school functioning (5-items). This questionnaire has excellent psychometric properties in pediatric cancer with Cronbach's alpha coefficients for the total scale score of α = 0.88 for child self‐report and α = 0.93 for parent proxy-report (7, 12).

**Detailed method for trametinib serum dosing**

Trametinib is measured in human plasma using a high performance liquid chromatography (HPLC) system (Series 1200 Infinity) coupled to a tandem mass spectrometer (MS/MS 6460), both from Agilent Technologies Inc, Canada. Plasma calibration samples and three plasma quality control samples were prepared by adding the appropriate amount of trametinib (Toronto Research Chemicals, Canada) to blank plasma, in accordance with the recommendations on bioanalytical methods validation stating that total added volume must be below 10% of the biological sample volume. Plasma aliquots (50 µL) were subjected to protein precipitation with 150 µL methanol containing the internal standard, trametinib-d4 (Santa Cruz Biotechnology, USA) and carefully vortex-mixed before being centrifuged. An aliquot (100 µL) of supernatant was transferred to an autosampler vial with glass insert. and 10 µL injected into the HPLC-MS/MS. Chromatographic separation was executed on a Phenomenex Kinetex® C18 2.6 μm (2.1 × 50 mm). Each injected sample was separated and eluted using a mobile phase gradient (10 mM ammonium acetate in water/ 0·1% formic acid : 10 mM ammonium acetate in methanol/ 0.1% formic acid) at a flow rate of 250 μL/min with a total run time of less than 8 min/sample. Detection was performed under positive-electrospray-ion multiple reaction monitoring mode using 616.1 → 491.0 transition for trametinib (Toronto Research Chemicals, Canada) and 620.1 → 495.0 transition for trametinib-d4. Validation of the method in terms of selectivity, accuracy, precision, recovery and stability was carried out in accordance with bioanalytical FDA and EMA guidances. Trametinib calibration curves were linear at concentrations ranging from 0.2 to 50 ng/ml with correlation coefficients r2 > 0.999.

**Data A3:**

**Additional information on two patients that received prior MEK inhibitor**

One patient was treated with trametinib for 18 months for an orbital and neck plexiform neurofibroma. The lesion was considered stable by the local investigator based on one-dimensional measurements. Two and a half years later, the tumor progressed on local assessment, and the patient elected to restart treatment. He subsequently achieved a partial response based on volumetric evaluation.

The other patient was initially treated with trametinib for weakness, pain, and increasing tumor bulk. Pain improved, with a mild reduction in the size of a large diffuse plexiform neurofibroma. Trametinib was discontinued after six months because of side effects, including stomatitis and paronychia. One year later, the patient restarted trametinib on study and experienced improvement in pain with tumor shrinkage (stable disease by volumetric evaluation). He later developed pneumonitis and discontinued treatment on study. Hypersensitivity pneumonitis due to avian antigen exposure was considered the most likely diagnosis, although a contributory role of trametinib could not be excluded.

**Figure A1**

**Figure A2**

a)

b)

c)

**FIGURE A2**

Relative change from baseline in % for patients a) < 3 years old b) ≥3 to <12 years old c) ≥12 years old

**Figure A3**

**Figure A3.**

Progression free survival of patients with NF1 and plexiform neurofibromas. Progression was defined has an increase of more than 20% in volume compared to baseline.

**Figure A4**

**Figure A4.**

Progression free survival of patients with NF1 and plexiform neurofibromas. Progression was defined has an increase of more than 20% in volume compared to smallest measured lesion.

**Table A2: Table of modified RECIST 1·1 response compared to volumetric assessment.**

| **Modified RECIST 1·1 overall response (CR/PR/MR) during treatment compared to Baseline** | **Volumetric overall response (CR/PR) during treatment compared to Baseline** | | |
| --- | --- | --- | --- |
| **Frequency (n) Percent (%)** | **No Response** | **Response** | **Total** |
| **No Response** | 13 28.3% | 8 17.4% | 21 45.7% |
| **Response** | 11 23.9% | 14 30.4% | 25 54·.4% |
| **Total** | 24 52.2% | 22 47.8% | 46 100% |

Concordance (green-58.7%) and discordance (red-41.3%) between one dimension assessment (modified RECIST 1.1) and volumetric assessment. CR : complete response, PR :partial response and MR: minor response**.**

**Table 3A. Treatment-Related Adverse Events**

|  | | | |
| --- | --- | --- | --- |
| **Subjects with at least one AE with reasonable possibility to be related to study treatment** | **NF1 with PN (N=46)** | | |
| **_** | **n (%)** | **Grade 1 or 2** | **Grade 3 or 4** |
| **GASTROINTESTINAL DISORDERS** |  |  |  |
| ABDOMINAL PAIN | 14 (30.4%) | 14 | 0 |
| CONSTIPATION | 11 (23.9%) | 11 | 0 |
| DIARRHEA | 23 (50.0%) | 23 | 0 |
| MUCOSITIS ORAL | 12 (26.1%) | 10 | 2 |
| NAUSEA | 14 (30.4%) | 14 | 0 |
| VOMITING | 13 (28.3%) | 13 | 0 |
|  |  |  |  |
| **GENERAL DISORDERS AND ADMINISTRATION SITE CONDITIONS** |  |  |  |
| FATIGUE | 15 (32.6%) | 15 | 0 |
| FEVER | 9 (19.6%) | 9 | 0 |
|  |  |  |  |
| **INFECTIONS AND INFESTATIONS** |  |  |  |
| FOLLICULITIS | 9 (19.6%) | 9 | 0 |
| PARONYCHIA | 30 (65.2%) | 29 | 1 |
|  |  |  |  |
|  |  |  |  |
| **INVESTIGATIONS** |  |  |  |
| ASPARTATE AMINOTRANSFERASE INCREASED | 3 (6.5%) | 3 | 0 |
| CPK INCREASED | 12 (26.1%) | 10 | 2 |
| EJECTION FRACTION DECREASED | 5 (10.9%) | 5 | 0 |
| NEUTROPHIL COUNT DECREASED | 3 (6.5%) | 1 | 2 |
| WEIGHT GAIN | 3 (6.5%) | 3 | 0 |
|  |  |  |  |
| **METABOLISM AND NUTRITION DISORDERS** |  |  |  |
| ANOREXIA | 5 (10.9%) | 5 | 0 |
|  |  |  |  |
| **NERVOUS SYSTEM DISORDERS** |  |  |  |
| HEADACHE | 5 (10.9%) | 5 | 0 |
|  |  |  |  |
| **RESPIRATORY, THORACIC AND MEDIASTINAL DISORDERS** |  |  |  |
| EPISTAXIS | 8 (17.4%) | 8 | 0 |
| PNEUMONITIS | 1 (2.2%) | 0 | 1 |
|  |  |  |  |
| **SKIN AND SUBCUTANEOUS TISSUE DISORDERS** |  |  |  |
| ALOPECIA | 23 (50.0%) | 23 | 0 |
| DERMATITIS | 7 (15.2%) | 7 | 0 |
| DRY LIPS | 4 (8.7%) | 4 | 0 |
| DRY SKIN | 24 (52.2%) | 24 | 0 |
| ECZEMA | 18 (39.1%) | 17 | 1 |
| ERYTHEMA | 4 (8.7%) | 4 | 0 |
| HAIR COLOR CHANGES | 17 (37.0%) | 17 | 0 |
| RASH | 3 (6.5%) | 3 | 0 |
| RASH ACNEIFORM | 21 (45.7%) | 20 | 1 |
| RASH MACULO-PAPULAR | 10 (21.7%) | 10 | 0 |
| SCALP PAIN | 3 (6.5%) | 3 | 0 |
| SKIN RASH | 4 (8.7%) | 4 | 0 |
|  |  |  |  |
| **VASCULAR DISORDERS** |  |  |  |
| HYPERTENSION | 7 (15.2%) | 6 | 1 |
|  |  |  |  |
| Treatment-Related Adverse Events (AEs) (By grade: 1, 2 or 3, 4). Only AEs with over 5% (≥ 3 patients) or with grade ≥3 are reported in this table. Only the occurrence with maximum grade is reported. | | | |
|  | | | |

**Table A4: Trametinib dose reduction due to AEs**

| **Patient #** | **Visit** | **Description of adverse event** |
| --- | --- | --- |
| 1 | Cycle 1 | Acneiform rash |
| 2 | Cycle 4 | Acneiform rash |
| 3 | Cycle 6 | Acneiform rash |
| 4 | Cycle 12 | Acneiform rash |
| 5 | Cycle 8 | Paronychia |
| 6 | Cycle 12 | Paronychia |
| 7 | Cycle 13 | Paronychia |
| 8 | Cycle 16 | Anemia |
| 9 | Cycle 9 | Hypertension |
| 10 | Cycle 12 | Weight gain |
| 11 | Cycle 9 | Cranial VI nerve palsy |
| 12 | Cycle 2 | Mucositis oral |
| 13 | Cycle 12 | Oral mucositis |
| 14 | Cycle 4 | Elevated creatine phosphokinase |
| 15 | Cycle 2 | Neutrophil count decrease |

**Table A5. Descriptive analyses of PedsQL brain tumor module and generic core scales for parent proxy‐report by subscale.**

|  | **N** | **BASELINE** | **CYCLE 7** | **CYCLE 13** | **END OF**  **TREATMENTS** | **F** | **p-value** | **ηp2** |
| --- | --- | --- | --- | --- | --- | --- | --- | --- |
|  |  | **Mean (SD)** | **Mean (SD)** | **Mean (SD)** | **Mean (SD)** |  |  |  |
| **PedsQL Brain Tumor module** |  |  |  |  |  |  |  |  |
| Cognitive problems | 29 | 63.81 (19.63) | 66.70 (25.02) | 70.94 (21.85) | 64.73 (23.44) | 2.668 | 0.069 | 0.235 |
| Pain & hurt | 35 | 77.38 (20.77) | 74.76 (17.79) | 77.02 (18.73) | 78.10 (17.63) | 0.549 | 0.652 | 0.049 |
| Movement & balance | 35 | 82.62 (21.33) | 79.52 (17.89) | 83.10 (19.75) | 82.5à (18.82) | 0.702 | 0.558 | 0.062 |
| Procedural anxiety | 35 | 69.05 (30.82) | 71.90 (28.23) | 80.00 (30.53) | 66.43 (30.88) | 2.673 | 0.064 | 0.200 |
| Nausea | 35 | 91.43 (15.37) | 87.21 (12.41) | 87.64 (17.14) | 88.86 (13.78) | 0.908 | 0.448 | 0.078 |
| Worry | 35 | 88.81 (22.14) | 74.76 (24.69) | 79.88 (20.92) | 78.81 (22.99) | 4.012 | 0.016 | 0.273 |
| Overall score | 35 | 79.70 (15.68) | 76.33 (14.69) | 80.21 (16.31) | 77.18 (15.33) | 1.997 | 0.134 | 0.158 |
| **PedsQL generic core** | | |  |  |  |  |  |  |
| Physical functioning | 38 | 76.60 (22.74) | 73.65 (22.05) | 78.38 (18.47) | 76.32 (21.32) | 1.075 | 0.372 | 0.084 |
| Emotional functioning | 38 | 70.89 (18.49) | 70.82 (18.23) | 72.24 (20.19) | 69.51 (18.78) | 0.321 | 0.810 | 0.027 |
| Social functioning | 38 | 77.54 (21.14) | 75.10 (20.03) | 80.79 (18.94) | 76.58 (22.69) | 1.380 | 0.265 | 0.106 |
| School functioning | 37 | 61.55 (18.42) | 63.03 (17.69) | 63.1à (18.44) | 63.69 (24.09) | 0.125 | 0.945 | 0.011 |
| Overall score | 38 | 71.76 (16.17) | 70.73 (15.60) | 73.74 (15.96) | 71.61 (18.35) | 0.912 | 0.445 | 0.073 |
